# Embryonic origins of human vascular smooth muscle cells: implications for in vitro modeling and clinical application

**DOI:** 10.1007/s00018-013-1554-3

**Published:** 2014-01-18

**Authors:** Sanjay Sinha, Dharini Iyer, Alessandra Granata

**Affiliations:** 1 Anne McLaren Laboratory for Regenerative Medicine, University of Cambridge, Cambridge, CB2 0SZ UK; 2 Wellcome Trust-Medical Research Council Cambridge Stem Cell Institute, Tennis Court Road, Cambridge, CB2 1QR UK

**Keywords:** Smooth muscle cell, Embryo, Vascular disease, Disease modeling, Stem cell

## Abstract

Vascular smooth muscle cells (SMCs) arise from multiple origins during development, raising the possibility that differences in embryological origins between SMCs could contribute to site-specific localization of vascular diseases. In this review, we first examine the developmental pathways and embryological origins of vascular SMCs and then discuss in vitro strategies for deriving SMCs from human embryonic stem cells (ESCs) and induced pluripotent stem cells (iPSCs). We then review in detail the potential for vascular disease modeling using iPSC-derived SMCs and consider the pathological implications of heterogeneous embryonic origins. Finally, we touch upon the role of human ESC-derived SMCs in therapeutic revascularization and the challenges remaining before regenerative medicine using ESC- or iPSC-derived cells comes of age.

## Introduction

The propensity for vascular diseases involving smooth muscle cells (SMCs) such as atherosclerosis and aortic aneurysms to present at specific vascular locations and territories has long been thought to be a function of hemodynamics and underlying vessel structure. However, there is increasing evidence that SMC embryonic lineage may also play a role in determining the location and presentation of disease. Vascular SMCs provide contractile function and structural support to blood vessels. Unlike other myocytes, vascular SMCs are not terminally differentiated and display remarkable phenotypic plasticity [1]. In healthy adult vessels, they proliferate at an extremely low rate and express a unique repertoire of contractile proteins. However, in response to local environmental cues, such as vessel injury, they are able to undergo changes in phenotype including down-regulation of contractile genes, increased proliferation, and remodeling of the extra-cellular matrix facilitating increased cell migration. Some of these SMC phenotypic changes are thought to contribute to or predispose to vascular diseases [2].

Interestingly, although many risk factors such as hyperlipidemia, diabetes, and hypertension are systemic, distinct vascular regions frequently display differential disease susceptibility or resistance [3, 4]. In addition to local factors such as blood flow, shear stress, and vessel wall composition, it has been proposed that vascular SMC embryonic origin influences disease localization and progression. A wide range of lineage tracking studies have shown that vascular SMCs arise from multiple different origins during development [5], raising the possibility that differences in embryological origins between SMCs could contribute to site-specific localization of vascular diseases such as aortic aneurysm [6], vascular calcification [7], and regional susceptibility to atherosclerosis [8, 9]. The potential importance of SMC lineage diversity in influencing vascular disease patterns as reported in these studies raises a number of fundamental questions. How do different embryonic tissues give rise to distinct SMC subtypes? What are the intrinsic differences between vascular SMCs of different embryological origins? Do vascular SMCs display origin-specific differences in response to disease inducing stimuli? These questions can only be fully addressed by generating in vitro and in vivo models of origin-specific SMCs.

During early vertebrate embryogenesis, the development of the vascular system is regulated by endothelial cells, SMCs, and pericytes. The organization of endothelial cells into the primary vascular plexus is initiated shortly after gastrulation and marks the onset of vascular development. The endothelial vasculature is subsequently remodeled by recruitment of SMCs and pericytes to form a complex vascular system [10, 11]. Endothelial cells originate mainly from mesodermal progenitors [12]. Since hematopoietic cells and endothelial cells express common markers such as CD31, CD34, and vascular endothelium cadherin (CDH5), it has been proposed that they originate from a common progenitor, the hemangioblast [13]. Most vascular SMCs are also largely derived from various mesodermal lineages such as splanchnic mesoderm, lateral plate mesoderm, and somatic or paraxial mesoderm [5, 14], although an important subset originates from the neural crest [5, 15]. The ontogeny of pericytes is less clear in comparison to SMCs and endothelial cells. They are generally thought to be of mesenchymal origin, although transdifferentiation from endothelial cells has also been suggested [16]. Some develop along with vascular SMCs from a common precursor. For example, pericytes in the aorta and coronary vessels originate from the somitic mesoderm and epicardial mesothelium, respectively [17, 18].

In this review, we aim to shed light on the molecular basis of SMC lineage diversity and how it might contribute to site-specific distribution of vascular diseases. In the following section, we review the developmental pathways and embryological origins of vascular SMCs and also briefly discuss the development of pericytes. This review then summarizes and discusses in vitro strategies for deriving SMCs from human embryonic stem cells (ESCs) and induced pluripotent stem cells (iPSCs), collectively known as human pluripotent stem cells (PSCs), their role in site-specific progression of vascular disorders, and their potential for disease modeling and therapy.

## Development of vascular SMC precursors in the embryo

**Fig. 1:**
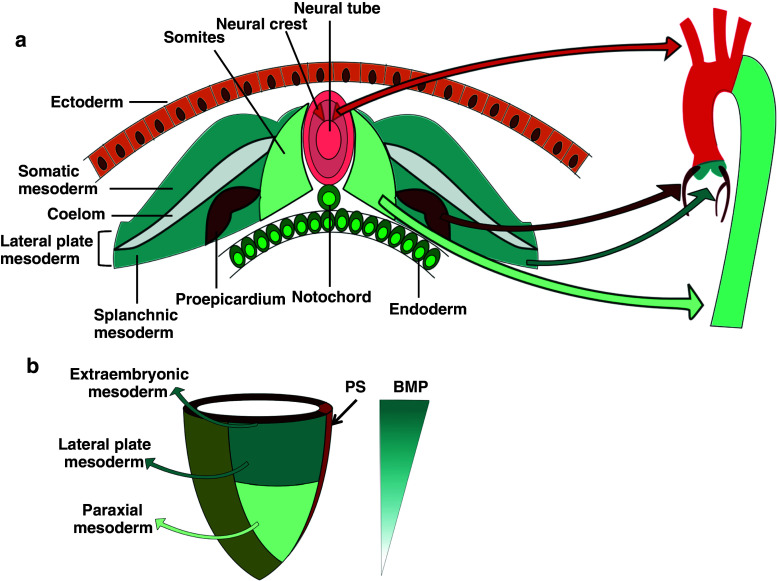
Distinct embryological origins for aortic SMCs and mesoderm patterning. **a** Schematic showing different embryonic tissues that contribute to SMCs in different vascular regions. Neural crest gives rise to SMCs in the ascending aorta and arch while the descending aorta is derived from the somites. The aortic root base originates from the secondary heart field, a lateral plate mesoderm derivative, while coronary SMCs arise from the proepicardium, also a lateral plate derivative. **b** Schematic of proximal–distal BMP gradient depicted in the murine E7.5 embryo. Local BMP concentration patterns cells emerging from the primitive streak into different mesoderm subtypes

The formation of the three germ layers, ectoderm, mesoderm, and endoderm, during the process of gastrulation is one of the defining events of embryogenesis. In mice, the pre-gastrulation epiblast is in the shape of a cup while the human equivalent is a flat disc. Gastrulation is marked by formation of the primitive streak in the posterior region of the epiblast at embryonic day 6.5 in mice [19] and at the beginning of the 3rd week in humans [20]. Cells in the most proximal portion of the primitive streak (in mice or in the most posterior region in man) undergo an epithelial to mesenchymal transition (EMT) to form the extra-embryonic mesoderm, while the mid and distal portions of the primitive streak give rise to embryonic mesoderm and endoderm, respectively. Vascular SMCs arise from multiple independent origins, and this topic has been reviewed in detail by Majesky [5]. Vascular SMCs of the ascending aorta, the aortic arch, and pulmonary trunk are neural crest-derived [15], whereas SMCs in the descending aorta originate from somitic mesoderm [14] (Fig. 1a). Fate-mapping studies have also shown that progenitors for coronary SMCs are found in the proepicardium, a transient structure in the looped heart stage that is located at the venous pole and originates from the lateral plate mesoderm [21]. In vivo studies in stage 14 chick embryos using lineage tracer dyes indicate that SMCs at the base of the aorta and pulmonary trunk originate from the secondary heart field [22, 23]. Other SMC precursors capable of differentiating into mature vascular SMCs include Nkx2-5^+^ and/or Isl1^+^ cardiovascular progenitors, mesoangioblasts, hemangioblasts, and neural crest precursors [24].

In recent years, pericytes have gained increasing attention as important regulators of vascular development, maturation, and remodeling. The similarities in contractile properties and marker expression between SMCs and pericytes have made identification of pericytes a challenge. The ontogeny of pericytes and the signaling pathways involved in the pericyte–endothelium interaction have been recently reviewed [25]. Pericytes in the brain [26] and thymus [27] have been reported to have a neural crest origin whereas pericytes in the gut [28], lung [29], and liver [30] have been mapped to a mesenchymal origin. Pericytes in the aorta share similarities with vascular SMCs: they originate from a common precursor, the somitic mesoderm [17]. In addition, pericytes are believed to be able to differentiate into SMCs and vice versa in conjunction with vessel growth and remodeling. Whether pericytes are an intermediate step in SMC differentiation or develop by a different pathway remains to be established.

The spatial and temporal segregation of cell fates during gastrulation suggests that different regions of the primitive streak constitute distinct signaling environments that are responsible for the induction of specific lineages. The developmental cues that trigger the various signaling pathways are usually transcription factors, epigenetic regulators, and cytokines. The signaling pathways involved in early embryonic development and cell lineage specification have been reviewed by Roper and Hamberger [31]. In the following section, we provide a brief overview of the cross-talk between signaling pathways in the induction of specific SMC precursor lineages.

### Development of the neural crest

**Fig. 2:**
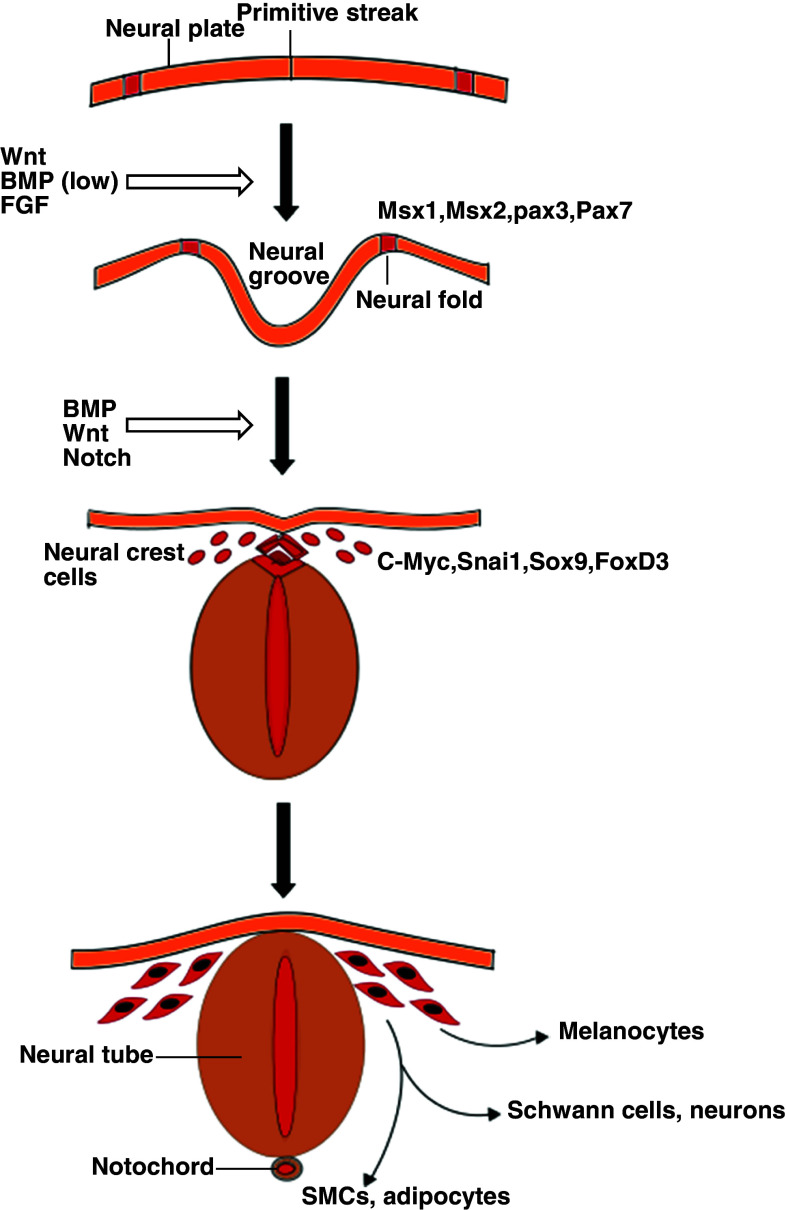
Neural crest development. Neural crest forms at the junction between neural plate and non-neural ectoderm. Inductive signals include Wnt, BMP, and FGF signaling. The neural plate border regions are specified by a range of transcription factors that include Msx1, Msx2, Pax3, and Pax7. Subsequently, a group of neural crest specifiers including c-Myc, Snai1, Sox9, and FoxD3 are expressed in migrating neural crest cells, which can self-renew or differentiate to a range of cell types

In the early stages of development, neural tissue is induced in the ectodermal layer of the embryo. Upon induction, the ectoderm gets separated into three different regions: (1) neural ectoderm gives rise to the nervous system; (2) non-neural ectoderm develops into the epidermis; and (3) neural crest cells at the border between the neural and non-neural ectoderm, contribute to the formation of peripheral neurons, glial cells, and SMCs of the ascending aorta, aortic arch, and pulmonary trunk [15, 32]. Neural crest cells are derived from delamination of the dorso-lateral neural tube beginning on day 8.5 of murine development [33] and at approximately the 4th week of human development [34]. The induction of neural crest precursors occurs at the edge of the neural plate and is regulated by multiple signaling pathways including bone morphogenetic protein (BMP), wingless-type MMTV integration family (Wnt), fibroblast growth factor (FGF), and notch [35, 36] (Fig. 2). Numerous studies have shown that diminished BMP signaling promotes neural induction during vertebrate development [37]. Other members of the transforming growth factor (TGF)-β family such as nodal have also been reported to inhibit the induction of neuroectoderm in vivo [38].

Critical insights into the genetic network regulating neural crest function and migration have been obtained from patients with genetic deletions affecting neural crest-derived tissues. For example, in DiGeorge syndrome, a chromosome 22q11 deletion is associated with conotruncal defects as well as palatal defects and a hypoplastic thalamus [39]. Mouse models of this condition have identified likely candidate genes such as *Tbx1* which lies in the deleted region and has a major non-cell autonomous role in regulating neural crest migration [40]. However, isolated functional mutations of *TBX1* have so far not been identified in patients with DiGeorge syndrome, suggesting that other genes and distal modifiers are important for the development of the full phenotype.

### Development of the mesoderm and its subtypes

Vascular cells including endothelial cells and SMCs are predominantly derived from the mesoderm lineage. The primitive streak is a key structural component that discriminates the mesodermal precursors. Developmental studies in *Xenopus laevis* have shown that cells migrate from the epiblast through the primitive streak and organize into the mesodermal germ layer [41]. The mesoderm subtypes, which include axial, paraxial, intermediate, and lateral plate mesoderm, are formed in order of their proximity to the primitive streak [42–44]. The patterning of mesoderm is influenced by multiple signaling gradients, growth factors, and transcriptional factors and is generally conserved across species [45]. Early in vivo studies in *Xenopus* and zebrafish embryos have shown that FGFs, Wnt, and members of the TGF-β family, which include the BMPs, activin, and nodal molecules, play important roles in the induction and patterning of mesoderm [46, 47]. Marginal zone patterning experiments in *Xenopus* embryos have also shown that a posterior to anterior BMP4 gradient gives rise to mesodermal subtypes. A higher concentration of BMP4 facilitates the formation of the lateral plate mesoderm while low concentrations give rise to paraxial mesoderm [48] (Fig. 1b). However, the precise functional relationship among these pathways in the induction and patterning of the mesoderm and its subtypes remains to be defined.

### Development of the proepicardium

Coronary SMCs lining the walls of the coronary arteries are an important class of SMCs that originate from the proepicardium. The proepicardium is a transient mesothelial structure found in the wall of the pericardial cavity between the sinus venosus and the liver primordium during development of the heart tube. The proepicardium gives rise to epicardium, the epithelial tissue covering the heart. Epicardial cells undergo EMT and invade the myocardium to become cells of the coronary vasculature [49, 50]. Although the importance of the proepicardium for heart development is clear, the signals that direct its formation are just beginning to be understood [51]. The proepicardium is believed to have its origin from the lateral plate mesoderm progenitors that express *Nkx2.5* and *Isl1* [52]. Early in vivo experiments in chick showed that a distinct level of BMP2 signaling is required for inducing proepicardium-specific gene expression [53]. Low levels of BMP2 induce/maintain proepicardium-specific gene expression whereas high levels promote myocardium formation. These findings also suggest that, although BMP is necessary, it is not sufficient for proepicardium induction and is likely to converge with other signaling molecules. In support of this, Kruithof and colleagues demonstrated that a cross-talk between FGF and BMP signaling is critical in determining a proepicardial fate [54]. Other signaling pathways that regulate epicardium and coronary vessel development include retinoic acid, Wnt, notch, and sonic hedgehog (SHH) [55]. What is not so well established is the cross-talk of various signaling pathways that direct epicardial differentiation to an endothelial, smooth muscle, or cardiomyocyte lineage. An alternative source of epicardial cells has also been described at the arterial pole, known as the arterial proepicardium, which gives rise to epicardial cells surrounding the intrapericardial segment of the great vessels [56]. While these cells are also able to undergo EMT and contribute to epicardial-derived cells in the outer layers of aortic and pulmonary arteries, the mechanisms regulating their distinct migratory and functional properties are less well characterized than for the better studied sinus venosus-derived epicardial cells that surround the majority of the myocardium. Besides understanding how the epicardium is formed, it is also important to identify the developmental signals that initiate proepicardium formation. Recent studies suggest that tissues lying in close proximity of the developing proepicardium, such as liver buds, promote proepicardial gene expression through localized inductive signals [57]. Nevertheless, further investigations on tissue interactions at earlier stages are necessary to identify new candidate signals that instruct cell fate during proepicardium development.

## In vitro models of early embryonic development

**Fig. 3:**
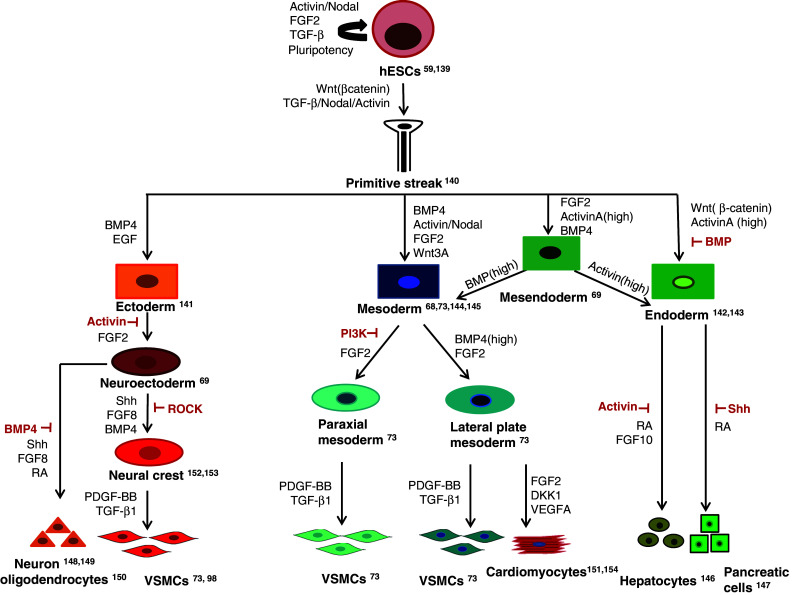
In vitro differentiation of human ESCs to embryonic intermediates and specific tissues. Some of the major in vitro differentiation steps are described using chemically defined conditions in hESCs. Inductive agents required for each step were based on developmental studies in non-human embryos. Factors inhibiting specific steps are shown in *red*. References to studies describing the generation of each cell type are provided in *superscript* [59, 68, 69, 73, 98, 139–154]. *EGF* epidermal growth factor, *BMP4* bone morphogenic protein-4, *TGF-β* transforming growth factor-β, *FGF* fibroblast growth factor, *Shh* sonic hedgehog, *PDGF-BB* platelet-derived growth factor-BB, *RA* retinoic acid, *VEGFA* vascular endothelial growth factor A, *DKK1* Dickkopf-related protein 1, *PI3K* phosphoinositide 3-kinase

Pluripotent human ESCs derived from the inner cell mass of the blastocyst are unique tools for studying early human embryonic development and differentiation in vitro as they are equivalent to an epiblast stage of commitment, open to all lineage pathways [58]. It is clear that understanding the regulation of pluripotency and early developmental events in human ESCs is a pre-requisite for directed differentiation into specific mature cells and tissues. With these aims in mind, many of the developmental paradigms reported in studies on human and non-human embryos have been used to establish chemically defined conditions for either maintaining pluripotency or early lineage specification of human ESCs in vitro (Fig. 3). From these studies, it has emerged that key signaling pathways such as activin/nodal, Wnt, insulin-like growth factor (IGF)-1, and FGF are important for maintaining the pluripotent capacity of human ESCs [59–62]. This contrasts with the signaling pathways required by murine ESCs which depend upon leukemia inhibitory factor (LIF) and BMP4 signaling for pluripotency [63]. However, this paradox may be explained by the recent discovery of murine epiblast stem cells [64, 65], which are the in vitro equivalents of the post-implantation epiblast and have similar signaling requirements to human ESCs for pluripotency. Thus, human ESCs are temporally dissociated from murine ESCs but may be equivalent to murine epiblast stem cells.

A variety of signals including BMP, nodal, Wnt, and FGF have been implicated in mesoderm development in vivo [46, 47]. These signaling factors exhibit extensive cross-talk in studies on embryological development which, along with the establishment of concentration gradients in the epiblast or primitive streak, lead to specific mesodermal patterning in the embryo. Key questions include which of these factors are required for mesoderm specification from human ESCs and how best to model the complex series of embryological events in vitro. Embryoid body-based studies clearly showed that almost all cardiovascular cell types could be generated in vitro along with the prior appearance of a wide variety of early embryonic tissues including mesoderm. However, the heterogeneous nature of embryoid bodies, frequent use of foetal bovine serum, and the difficulty of reliably getting reagents into the center of an embryoid body make it difficult to identify specific factors required for differentiation. Consequently, we will focus our review predominantly on chemically defined human systems with an emphasis on monolayer differentiation.

In view of the pressing problem of cardiomyocyte loss in patients suffering from ischemic heart disease, several groups have attempted to generate cardiomyocytes and cardiac mesoderm, a derivative of ventro-lateral or splanchnic mesoderm, from human ESCs. LaFlamme and colleagues [66] used sequential addition of activin A and BMP4 to induce cardiomyogenesis efficiently in a chemically defined monolayer system. Their rationale was to initially generate primitive streak-like cells with subsequent cardiomyocyte induction. However, while the protocol was relatively efficient at producing cardiomyocytes, the intermediate states were poorly characterized. Subsequently, Yang and Keller used a staged approach to model embryological events which involved activin A, BMP4, and FGF2 to establish a primitive streak-like population, then Dikkopf 1 homolog (DKK1) and vascular endothelial growth factor (VEGF) to induce cardiac mesoderm followed by the addition of FGF2 to expand the cardiomyocyte population [67]. They carefully defined different types of lateral plate mesoderm derivatives with either cardiac or hematopoietic/vascular developmental potential using flow sorting, but did not show the presence of an unpatterned lateral plate mesoderm population. Although the system was chemically defined, the use of embryoid bodies limits the reproducibility and scalability of this approach. Subsequently, it was shown that short-term addition of BMP4 along with the action of endogenous activin A and FGF led to the development of an early mesoderm population [68], while Vallier et al. [69] showed that addition of activin A, BMP4, and FGF2 led to the induction of mesendoderm in chemically defined monolayer systems. These studies also confirmed previous in vivo findings that FGF signaling was required in addition to activin to efficiently form mesoderm [46, 70].

Besides mesoderm, BMP signaling has also been reported to promote the expression of genes associated with trophoblast [71]. Among the selective markers utilized to define and track the patterning and induction of mesodermal cells in culture, brachyury (T) is one of the best markers of mesoderm differentiation and is widely used to track mesoderm induction. In a recent study, Bernado and colleagues showed that BMP4 and FGF2 via an Erk-mediated signal pathway cooperate to drive human ESC differentiation into mesodermal cells that express high levels of T [72]. The extensive characterization done in this study helps to answer the paradoxical question of whether the trophoblast-like culture induced by BMP [71] is an artefact or models embryonic development. Their findings reveal a new role for caudal type homeobox2 (*CDX2*) and other genes previously regarded as markers of trophoblast in human mesoderm development and places *T* upstream of these genes. Extending this work, we have recently shown that, by mimicking the embryonic BMP concentration gradient along the primitive streak, specific mesoderm subtypes—namely paraxial or lateral plate—can be induced from uncommitted mesoderm in vitro [73]. Human ESC-derived early mesoderm when treated with FGF2 and BMP4 for 5 days generated cells that expressed lateral plate mesoderm markers, whereas treatment with FGF2 and Ly294002 (a phosphoinositide-3 kinase inhibitor) in the absence of BMP4 was optimal for obtaining a population that expressed paraxial mesoderm markers.

The other major source of vascular SMCs is neural crest which is a neuroectodermal derivative. Development of neuroectoderm in vivo is induced by FGF2, Wnt, and inhibition of nodal and BMP [74]. Neuroectoderm appears to be the default differentiation pathway for ESCs when pluripotency factors are absent [75], and, consistent with these findings, Vallier and colleagues have shown that nodal signaling inhibits neuroectoderm specification during human ESC differentiation [76]. Furthermore, FGF2 and activin/nodal inhibition in vitro promote greater expression of neuroectodermal markers such as SRY-related HMG-box (SOX)1, SOX2, gastrulation brain homeobox (GBX)2, and nestin [69, 77]. A similar approach in inhibiting both activin/nodal and BMP in the presence of FGF2 gave rise to early neuroectoderm that developed into either neuronal tissue or neural crest. Although many investigators have focused on neuronal development, several protocols have also been described for induction of neural crest from human ESCs [78].

In most of these in vitro methods, the distinction between neuroectoderm, mesoderm, endoderm, and their subsequent progeny is based solely on the relative expression of marker genes. This could be of concern, as a large number of genes are shared between the embryonic lineages instead of being lineage-specific. One approach may be to link key transcription factor pathways with epigenetic changes during early differentiation of human ESCs, which may facilitate better discrimination between different lineages and also enable more efficient differentiation of ESCs towards specific vascular cell types. Alternatively, transcription factor combinations may prove to be more specific than single markers alone, while fluorescent reporters based on key lineage-specific genes could facilitate sorting of subtypes [79, 80]. Importantly, identification of specific surface markers [81] would also permit better selection of key populations that would be amenable to later translational applications. Consequently, development of such reporter lines and novel cell surface lineage-specific markers is urgently required to facilitate the optimization of differentiation protocols and isolation of different vascular cell lineages.

## Generation of human embryonic stem cell-derived smooth muscle cells

In their normal environment in vivo, healthy, and mature vascular SMCs are relatively easily identified by their anatomical location, in addition to a range of other phenotypic features. However, given the absence of anatomical cues in culture, it is essential that we carefully define the characteristics of this cell type before we can consider methods for its generation in vitro. Mature SMCs typically express a range of characteristic markers including smooth muscle alpha actin (ACTA2), SM22α (TAGLN), h1-calponin (CNN1), smoothelin (SMTN), and smooth muscle myosin heavy chain (MYH11), and possess a typical ultrastructural appearance involving a well-developed contractile apparatus and focal adhesions. However, defining a SMC in vitro is made challenging since many accepted SMC markers are also expressed, at least transiently, by other cell types, particularly during development or disease. For example, ACTA2, which is commonly used by many authors to denote SMCs, is also expressed by cardiomyocytes and skeletal myoblasts during normal development, by activated fibroblasts in wound repair, and by some tumor cells [82]. Perhaps the two most specific markers of the SMC lineage are SMTN and MYH11, and, for identification in vitro, it is important that expression of at least one of these genes is demonstrated in addition to a range of other characteristic markers. It should also be remembered that a key functional characteristic of a mature SMC is its ability to modulate calcium transients and contract in response to agonists. Thus, to stringently define a mature SMC in vitro, contractile ability also needs to be demonstrated. However, expression of SMTN and MYH11 and contractile ability are only acquired late in development, and these characteristics are lost early on in disease or following serum stimulation, thus complicating precise identification of cells in vitro or indeed in disease models in vivo.

A variety of in vitro models for human SMC differentiation have been described [24, 83–85]. These approaches generally build upon one or more strategies already established in mouse ES cells: (1) embryoid body formation followed by treatment with growth factors; (2) sorting for cell surface markers using fluorescence-activated cell sorting (FACS) or magnetic activated cell sorting (MACS) followed by differentiation; (3) culture on polymer coatings [Type IV collagen, fibronectin (FN1), gelatin] and on feeder cells; and (4) growth in the presence of soluble growth factors such as platelet-derived growth factor-BB (PDGF-BB) and TGF-β. A major advantage of the embryoid body method is that the molecular mechanisms of SMC differentiation may be studied in an environment that recapitulates early embryonic development. The use of extra-cellular matrix (ECM)-coated media such as collagen Type IV helps to mimic the microenvironment found within mammalian tissues and has been demonstrated to drive stem cell differentiation towards functional SMCs [86, 87]. Similarly, isolation of positive and negative progenitor populations based on expression of cell surface markers is an efficient way to induce differentiation selectively to a SMC or endothelial lineage [83]. However, there are limitations to some of these approaches. First, addition of growth factors to the culture medium may only be fully effective for the cells on the exterior of embryoid bodies that are in direct contact with the medium, since there is evidence that tight cell–cell junctions and extracellular matrix limit diffusion of substances into the center of the embryoid body [88]. Secondly, differentiation is remarkably heterogeneous in embryoid bodies limiting the efficiency of SMC differentiation and making it difficult to separate them from other cell types. Finally, sorting by FACS reduces the viability of recovered cells and has the risk of introducing intermediate stages during the differentiation process. Several advances, such as adherent monolayer differentiation system, use of defined serum-free media with specific inducers, and genetically modified reporter ESCs, have proven effective in overcoming these challenges [84, 89–91].

Despite the progress in generating human SMCs in vitro [24, 92, 93], in the majority of cases it is unclear which embryonic lineage is represented by the resulting cells. More importantly, until recently there was no attempt to determine whether human ESC-derived SMCs of different lineages behaved differently from each other, as has previously been shown using SMCs from avian or murine vessels [94, 95], and whether this had any significance for the development of disease. In vitro strategies for generating early embryonic lineages as discussed in the previous section offer the possibility of obtaining lineage-specific vascular SMCs for potential applications in regenerative medicine. We recently reported a chemically defined method for generating origin-specific vascular SMCs from human PSCs through the intermediate lineages—neuroectoderm, lateral plate mesoderm, and paraxial mesoderm [73]—which represent the embryonic origins of the majority of vascular SMCs. Intermediate lineages treated with PDGF-BB and TGF-β1 for 12 days resulted in differentiated vascular SMCs that expressed SMC markers and displayed contractile function. Validation studies, such as a specific requirement for myocardin-related transcription factor-B (MKL2) for vascular SMC differentiation from neuroectoderm and increased proliferation of neuroectoderm-derived SMCs when exposed to anigotensin II or TGF-β1, recapitulated previously described differences between distinct vascular SMC populations in vivo [94, 95]. These data suggest that the SMCs produced are in all likelihood origin-specific. To date, the study of origin-specific differences in human vascular SMCs and the implications for disease development have been limited by the practical difficulties associated with obtaining sufficient quantities of healthy human SMCs from a variety of anatomical locations. Our recent in vitro model offers a way to generate large numbers of lineage-specific vascular SMC subtypes with high efficiency from a single pluripotent source that may be used for comparative studies on the effects of lineage on disease development.

Immortalized primary neural crest stem cell (NCSC) lines have long been used as a model to study SMC differentiation in vitro [96]. When treated with TGF-β, NCSCs have been shown to be capable of inducing SMC marker genes [97]. Recently, Wang and colleagues have reported a method of obtaining SMCs from NCSCs derived from human ESCs and iPSCs [98]. Rosette structures developed from human embryoid bodies and cultured in neural crest medium formed neural sphere-like aggregates that expressed NCSC markers such as nestin, vimentin, and beta-1,3-glucuronyltransferase 1 (B3GAT1) and could be induced to neuronal and mesenchymal lineages. Treatment with TGF-β1 for 2 weeks induced differentiation of NCSCs into a SMC lineage expressing SMC markers. While the NCSCs have been well characterized in this work, further studies in addition to immunohistochemical analysis are required to fully characterize the neural crest-derived SMCs.

Despite the ability to generate lineage-specific SMCs as presented above (and summarized in Fig. 3), much further work remains to be done. There is great potential for refining these methods to generate more specific subtypes of smooth muscle and mural cells. For example, deriving coronary artery SMCs would facilitate further studies on the differences between atherosclerosis in this critical vascular bed compared to other vascular territories. Recently, El-Mounayri and colleagues [99] described a serum-free method of generating SMCs using directed differentiation of embryoid bodies towards a cardiac fate. The derived SMCs were deemed ‘coronary’ by the similarity of their Ca^2+^ and contractile responses to coronary SMC controls. However, this is not a highly specific test and, given their cardiac mesoderm developmental fate, the cells could alternatively be equivalent to aortic root SMCs. Further studies including extensive gene expression analyses may be required to validate their precise subtype. An alternative approach would be to use our knowledge of specific factors regulating epicardial development from splanchnic mesoderm as summarised previously in “In vitro models of early embryonic development”, in combination with a stepwise lineage selection approach to generate epicardium prior to SMC derivation. Another cell type of high clinical importance is the pericyte. While available evidence suggests common developmental origins for pericytes and SMCs in the same vascular bed [17, 18, 24], at some point the development of pericytes must diverge from their closely related SMCs. Methods to direct differentiating embryonic tissues into a pericytic rather than SMC fate would be invaluable for the study and the reconstruction of the micro-vasculature.

In a recent report, Dar and colleagues describe a method of isolating pericyte progenitors alongside endothelial cells and SMCs from differentiating embryoid bodies on the basis of the vascular cell markers, CD105 and CD31, using MACS [100]. The strength of this model lies in the robust characterization studies performed to ascertain that the novel subset (CD105^+^CD31^−^) of cells isolated are genuine pericytes. These cells emerge spontaneously within differentiating embryoid bodies, express commonly accepted antigenic markers of pericytes, and exhibit robust vasculogenic potential both in vitro and in vivo. One of the limitations of this model lies in failing to address the developmental origins of the pericytes. However, a combination of a lineage specific approach with the markers and methods used in this study may facilitate the emergence of lineage-specific pericytes.

Taken together, the current in vitro models are powerful tools to study SMC differentiation and maturation in a controlled environment. The pure populations obtained from these methods have greatly facilitated the biochemical characterization of SMCs derived from human ESCs. The ease of genetic manipulation of human ESCs makes this an extremely valuable tool for monitoring cell fate during different stages of differentiation [101]. Genetic selection of cells based on the expression of a selectable marker driven by lineage-specific promoters offers increased purity and excellent scalability for applications in regenerative medicine. However, there remain significant scientific obstacles with the current differentiation protocols that must be resolved. First, the time frame for SMC differentiation reported so far is remarkably short compared to the extended maturation phase in developing embryos, raising the possibility that there could be intermediate steps that further need to be modeled, or the need for extended culture to obtain fully mature vascular SMCs. Second, it is unclear whether the current models recapitulate a large number of developmental cues, such as microRNA signaling, epigenetic modifications, and histone deacetylase signaling, which normally guide differentiation and development of vascular SMCs in vivo. Moreover, environmental cues such as heterotypic cell–cell interactions, blood flow, and wall stress are not replicated in most in vitro systems. Finally, the markers commonly used to define SMC phenotype such as ACTA2, MYH11, and CNN1 are common to all SMC subtypes and do not identify the lineage of origin. Similarly, pericytes and SMCs co-express many markers. Studies aiming to identify SMC lineage-specific and pericyte-specific markers would help to distinguish between SMC subtypes and pericytes and are urgently needed.

## SMC disease modeling using iPSCs

There are several major challenges to understanding the detailed pathophysiology of vascular diseases. First, there are practical issues with obtaining human tissues. Surgical specimens usually represent end-stage disease, making it difficult to identify the initiators of disease or to delineate cause and effect. Next, mouse models, particularly genetically modified versions, have been extremely useful for studies into a wide variety of vascular diseases including atherosclerosis and aortic aneurysms, and have been reviewed in detail in several recent publications [102–104]. However, despite their benefits, there remain significant limitations when modeling human diseases due to many factors including disparities in vessel size, species-specific differences in metabolic and biochemical activity, and underlying differences in chromosomal and genomic organization. A highly pertinent example is that mouse models of atherosclerosis such as the apolipoprotein E (*ApoE*)-null or low density lipoprotein receptor (*Ldlr*)-null mice on high fat diets develop high grade atherosclerotic lesions but only poorly model plaque rupture, a key event in advanced human disease. Finally, many vascular diseases have a characteristic distribution or location despite systemic risk factors such as hypertension, lipid levels, or diabetes, which has in the main been attributed to anatomical and hemodynamic factors. However, SMCs display a great diversity of embryonic origins in-between blood vessels of different organs and sometimes even within the same blood vessel. Consistent with their different developmental origins, vascular SMCs show differences in growth, transcriptional responses to pleiotropic cytokines, such as TGF-β1, functional properties, and response to environmental cues [94, 105, 106]. These factors pose a critically important question: what is the significance of SMC lineage diversity for the site-specific localization of adult vascular diseases? In this section, we will review the potential for vascular disease modeling using iPSC-derived SMCs and consider the implications of heterogeneous embryonic origins.

Human iPSC-based systems, generated by reprogramming patient-derived somatic cells into pluripotent stem cells [107], have great potential for investigating disease pathophysiology by providing a parallel human system to complement mouse models. Of crucial importance, a lineage-specific model of vascular SMC development may be used to determine the role of embryonic origin in disease and provide the correct sub-type of SMC for accurate disease modeling in vitro. A major advantage of using patient-derived iPSCs, when modeling genetic diseases is that the resultant SMCs not only contain the disease causing mutation but also have the permissive genetic background required in many cases for full expression of the disease. Studies using iPSCs to model Hutchinson-Gilford progeria (HGP) syndrome and elastin deficiency have been recently published [108–110]. In HGP syndrome, a Lamin A (*LMNA*) mutation leads to accumulation of the mutant protein progerin and predisposes to increased DNA damage. Using HGP iPSCs, vascular SMCs were found to be among the most severely affected cell types, which may account for the accelerated atherosclerosis seen in this condition [109]. In a similar study, progerin was found to bind to the DNA-dependent protein kinase catalytic subunit which reduced the nuclear holoenzyme and resulted in decreased SMC proliferation [108]. Recently, Ge and colleagues modeled elastin deficiency which leads to supravalvular aortic stenosis [110]. SMCs from patient-derived iPSCs showed increased proliferation and migration that was due to increased ERK1/2 activity. These studies highlight how iPSC-based in vitro models may be used to generate new insights into the molecular mechanisms underlying their respective diseases. Many aspects of the molecular pathology still need to be clarified in both of these conditions, and the iPSC-based models offer a complementary system in human cells alongside established mouse models. However, despite these promising initial results with iPSC-based SMC disease modeling, several key issues need to be considered [111]. First, multiple iPSC clones are generally derived from each patient during reprogramming. Since individual lines, even from the same donor, may vary in their ability to generate somatic tissues and their subsequent phenotype [112], then which line or lines should be used for the disease-modeling studies? Another key question is the nature of the wild-type or negative controls. Are age- and sex-matched wild-type iPSCs sufficient? Should investigators use sibling-derived iPSCs or is it necessary to correct the genetic defect in the patient-derived iPSCs and show resolution of the disease phenotype? Bearing in mind these caveats, we now examine other vascular diseases that may also benefit from a human iPSC-based in vitro model, and expand on the utility of a lineage-specific approach for these investigations.

In the blood vessel wall, SMCs produce a complex ECM which is responsible for the mechanical properties of the wall. The ECM is subjected to proteolytic degradation which results from a balance between matrix metalloproteinases (MMPs), and their physiological inhibitors (TIMPs). Some of these proteases may be involved in some level of tissue reconstruction and/or remodeling. However, in the case of vascular diseases, such as aneurysms and atherosclerosis, the main role assigned to MMPs is matrix degradation, which causes weakening of the arterial wall that in the aorta can lead to dilatation, tortuosity, dissection, and rupture [113, 114]. Interestingly, the site of the initial dissection frequently occurs in regions where SMCs from two different embryonic origins are juxtaposed, near the aortic root or at the aortic isthmus. We recently showed that human SMCs of different embryonic lineages differed significantly in their ability to produce MMPs and TIMPs, which could lead to a step change in ECM degradation and mechanical properties at the junctions, possibly accounting in part for the increased likelihood of dissection at these sites [73]. Further studies, perhaps using lineage-specific SMC co-cultures, are required to test this intriguing hypothesis.

Marfan syndrome is caused by mutations in the gene encoding fibrillin1 (FBN1), an essential matrix protein. FBN1 both provides a scaffold for the assembly of microfibrils and elastic fibers and regulates the signaling events that occur between cells and the ECM [115] by sequestering TGF-β and BMP in the extracellular matrix. Since several elastin-deficiency states do not exhibit aortic aneurysm as a predominant phenotype [103, 116], it is likely that Marfan aortic aneurysms are not entirely caused by abnormal deposition of the elastic fibers. A range of studies including genetically modified mouse models have shown that, in response to FBN1 mutation, fibrillin1 protein shows an increased susceptibility to proteolysis, and that increased release of TGF-β may be a critical feature of this disease along with MMP upregulation [117, 118]. Studies in the mouse suggest that non-canonical TGF-β signaling pathways promote aneurysm development [119], but these observations need confirmation in a human system. Importantly, the aneurysm in Marfan syndrome develops preferentially in the ascending aorta and arch, regions that develop from neural crest and perhaps secondary heart field. A Marfan-iPS-based model of SMC development would facilitate investigations into the pathophysiology in a human system and would provide a test bed for novel therapies. Of key importance, the distinctive anatomical localization emphasizes the need for a lineage-specific in vitro model so that the correct sub-type of SMC could be used. It should be noted that the increase in non-canonical TGF-β signaling in the mouse model was only detected in the ascending aorta [120]. Indeed, it has been suggested that it may be the co-existence of two different sub-types next to each other which promotes the pathology seen in Marfans [103].

While Marfan syndrome is a good example of how a lineage-specific SMC developmental system would benefit investigation of a disease, other conditions in which SMCs display the primary pathology may profit from a similar approach. A similar aneurysmal pathology to Marfans is seen in Loeys–Dietz syndrome, which is associated with TGF-β receptor (TGFBR) mutations [121]. Interestingly, as with Marfan syndrome, there is evidence of increased TGF-β signaling, although the TGFBR mutations documented so far lead to loss of function. This paradox has yet to be resolved, and again a robust in vitro human system may be helpful. On the other hand, mutations in collagen III (*COL3A1*), another component of ECM, are known to cause vascular type IV Ehlers–Danlos syndrome (EDS), which gives rise to a more widespread pattern of aortic dissection compared to Marfans [122]. Since both collagen and fibrillin are ubiquitous ECM proteins, this divergence may be explained by possible differences in their contribution to the regulation of TGF-β. While the role of TGF-β in EDS is unknown, additional factors may influence the site of aneurysm development in patients affected by EDS. To identify these factors, an iPSC-SMC disease model for EDS, which may discriminate between the primary effect of *COL3A1* mutation and the involvement of other molecular pathways in the pathogenesis, could be generated.

Another set of mutations in encoding contractile proteins in vascular SMC, such as ACTA2 and MYH11, has been found to be responsible for isolated familial vascular disorder with ascending aortic aneurysm and dissection [123, 124]. This supports the idea that perturbation of SMC contractile apparatus is important for the pathogenesis of aneurysm. However, vascular SMCs isolated from patients with *MYH11* mutations have shown an upregulation of IGF-1 signaling and components of the angiotensin II cascade upstream of TGF-β [125]. This could imply that altered contractile properties are not the only mechanism responsible for site-specific aneurysm localization, and additional contributing factors related to SMC lineage diversity may be implicated.

An interesting example of how origin-specific SMCs could affect the localization of vascular pathology is cerebral autosomal dominant arteriopathy with subcortical infarcts and leukoencephalopathy (CADASIL), a cerebral arteriopathy accompanied by degeneration and loss of vascular SMCs [126]. CADASIL is caused by mutations in *NOTCH3*, which is exclusively expressed in SMCs and pericytes in the vessel wall. This results in the accumulation of the extracellular domain of NOTCH3 protein in the cytoplasmic membrane of SMCs, which contribute to the formation of pathognomonic granular osmiophillic material (GOM). Despite the characteristic ultrastructural appearance, the disease pathogenesis is still poorly understood. It is believed that CADASIL mutations interfere with normal cellular communication between SMCs and other components of the neuro-vasculature, for instance astrocytes. Although NOTCH3 is widely expressed in vascular SMCs throughout the body and pathognomonic GOM is seen in all arteries [127], SMC loss is restricted to the central nervous system [128], suggesting that neural crest-derived SMCs may be more susceptible to NOTCH3-compromised function than SMCs from other origins.

While much of this section so far has focused on conditions with a single gene disorder, there is potential for using iPSC- or ESC-derived SMCs to study more complex multi-genic disorders, including common conditions such as atherosclerosis. There is now accumulating evidence that SMC embryonic origin contributes to regional heterogeneity of disease as discussed in the introduction and earlier in this section. An unlimited source of lineage-specific human SMCs from PSCs, which correspond to disease-prone or disease-resistant vascular territories, would greatly facilitate studies on disease-causing mechanisms without confounding variables such as blood flow or challenges inherent in using diseased tissues in which cause and effect are difficult to disentangle. For example, we recently showed that atherosclerosis-prone aortic regions such as the aortic arch had lower levels of homeobox genes and higher levels of nuclear factor kappa-B (Nfkb1) activity than the atherosclerosis-resistant descending thoracic aorta [129]. HoxA9 and Nfkb1 displayed mutual inhibition and, importantly, the increased expression of *HoxA9* which reduced inflammatory activity in the descending aorta was also evident in corresponding human ESC-paraxial mesoderm-derived SMCs, thus establishing that regional differences in Hox gene expression were developmentally programmed. Building on these types of studies using human iPSC-derived cells would recapitulate the variable genetic background inherent in clinical diseases and provide a useful tool for further investigation of high-risk regions in the genome identified by recent large-scale genome-wide association studies, for example the 9p21 variant [130].

Despite these intriguing opportunities for vascular SMC disease modeling, a note of caution should be sounded. It is likely that in vitro conditions will not fully replicate the complexities of the in vivo milieu which include variables such as heterogeneous cell types, complex cell–matrix interactions, blood flow, pulsatile wall stretch, and systemic influences including circulating cytokines and growth factors. Careful phenotypic assessment of the disease models will be essential to determine to what extent the in vitro system is able to model the in vivo manifestations of the disease. Moreover, many of the diseases discussed in this section do not present until adulthood. Consequently, in vitro models may require long-term cultures or addition of factors that prematurely induce the disease phenotype.

In summary, iPSC-derived SMCs offer a novel system to study vascular disease in a human context that should complement existing techniques such as genetically modified mouse models. Bearing in mind the difficulty in sourcing primary adult vascular SMCs and their limited lifespan, key advantages include plentiful and reproducible quantities of human SMCs as well as the opportunity perhaps to study disease onset rather than the complex phenotypes seen in advanced disease. Finally, SMC functional differences which may be related to origin appear to play a fundamental role in pathophysiology of vascular disorders, emphasizing the need for lineage-specific model systems to study how these differences in SMC function may alter the onset and manifestation of disease.

## Regenerative medicine applications

Regenerating diseased tissues and organs, once thought to belong in the realms of science fiction, is now gaining widespread acceptance as one of the most exciting future applications of stem cell technology. Vascular regeneration has tremendous potential for restoring blood flow to ischemic tissues, either as an isolated strategy or in conjunction with regeneration of other cell types such as cardiomyocytes in the infarcted heart. Replacement of endothelial cells alone appears to be suboptimal, and the addition of mural cells/progenitors seems to enhance formation of a functional and enduring vasculature. We have recently reviewed the role of human ESC-derived SMCs in therapeutic revascularization [24] and so will only highlight the main issues in this section.

Clinical trials for cardiovascular regeneration to date have used adult stem cell populations such as bone marrow cells or mesenchymal stem cells with mixed results [131]. In these studies, there is little or no evidence for differentiation of transplanted progenitor cells into cardiomyocytes and only limited evidence for differentiation into vascular tissues; instead, the principal benefit is postulated to be paracrine in nature. These findings may reflect the limited plasticity of adult progenitors, and it is hypothesized that PSC-derived cells have a greater ability to form relevant tissues for vascular regenerative medicine. However, perhaps due to concerns over possible tumorogenicity, no clinical trials on vascular regeneration have yet been carried out using PSC-derived cells.

Despite these safety concerns, numerous pre-clinical studies have highlighted the tremendous regenerative potential of human PSC-derived vascular cells. The majority of studies have shown that ESC-derived vascular progenitors or cells can improve an ischemic hindlimb in rodent models [132, 133] and cardiac function in models of cardiac ischemia [134, 135]. Importantly, studies have also demonstrated the further benefit of transplanting mural cells over endothelial cells alone [87, 136]. However, the optimum combination of cell types remains to be determined, as does the degree of maturity of transplanted cells for maximum integration. It should be noted that, while studies using ESC-derived vascular cells do suggest some incorporation, to date there is a lack of detailed studies at single cell resolutions that rigorously quantify the extent to which transplanted cells directly contribute to the regenerative response. Given the opportunities available to genetically engineer reporter lines using ESCs, further studies documenting progenitor cell survival and differentiation in vivo using advanced imaging modalities can be envisaged and may help to determine the kinetics and course of vascular regeneration.

Although we have focused predominantly on SMCs in this review, therapeutic revascularization of the microvasculature will potentially require a pericyte-like cell, while SMCs may have more of a role in regeneration of larger vessels or ‘arteriogenesis’. As mentioned earlier, the origin of pericytes is still poorly established, although they may share similar developmental origins to SMCs in the same vascular bed. Protocols to generate pericytes from human ESCs in vitro have been described, although, given their phenotypic plasticity and the considerable overlap between them and SMCs, it is possible that it may be possible for them to interchange and switch phenotypes depending on external cues. For example, there are reports that pericytes may differentiate into SMCs, and we have previously demonstrated that a transient pericyte-like population is seen during SMC generation in vitro [73].

Many questions and challenges remain before regenerative medicine using ESC- or iPSC-derived cells comes of age. Concerns over possible tumorgenic side effects of ESC- or iPSC-derived vascular progenitors are paramount. However, new technologies to generate ‘integration-free’ iPSCs may address these concerns to some degree. Other key issues include whether it is more effective to use progenitor or differentiated cells? What stage of development and what combinations of cell types are optimal? Is there a requirement for exogenous ECM or growth factors to enhance the survival and development of the transplanted cells? Other regenerative medicine applications include the ex vivo generation of tissue-engineered vascular grafts for subsequent use by surgeons as grafts, and these have been generated from a variety of cell types including mouse ESC-derived cells [137]. Although similar structures have been engineered from human PSCs, these have not yet been tested in vivo [138]. Theoretically, there may be an advantage to using lineage-specific SMCs in a therapeutic context, for example, epicardial-derived SMCs for cardiac revascularization or artificial coronary bypass grafts. However, an alternative consideration may be to use SMC subtypes that maximally support endothelial network formation or those that display the most resistance to atherosclerosis. Studies that directly confirm an atherosclerosis-resistant SMC subtype are still required and could include the combination of in vitro responses of different SMC subtypes to inflammatory mediators, and the in vivo response to transplanting tissue-engineered grafts derived from different SMC sub-types into animal models of atherosclerosis. Extensive tissue-engineering studies will be required to optimize vascular regenerative therapies so that they will be suitable for the diverse clinical populations in need.

## Conclusions

This review has focused on the key steps of SMC embryonic development, highlighting the heterogeneity of vascular SMC origins. We have attempted to describe the recent progress in understanding the molecular and cellular pathways that contribute to the differentiation of SMC subtypes and the methods used to mimic this process in vitro. In particular, we have outlined the many possibilities for using human ESC- and iPSC-derived SMCs for disease modeling and potential therapeutic application. While the studies discussed in this review, and many others that could not be included due to space constraints, have made significant contributions to our understanding of vascular SMC development and disease, much remains to be done. For example, new lineage-specific in vitro models of SMC development offer an opportunity to test a long-standing question in developmental vascular biology—whether the heterogeneity of SMC origins contributes to the development and distribution of vascular disease. Other major challenges that may be amenable to suitable in vitro modeling include a detailed understanding of the SMC regulatory machinery in development and disease, and ways to translate our increasing biological knowledge into new therapeutic advances for patients. Nevertheless, the rapid progress in this field to date is a reflection of the synergy in bringing together the complementary fields of stem cell biology and vascular biology, and bodes well for further major advances.

## Abbreviations

BMP: Bone morphogenetic protein
CADASIL: Cerebral autosomal dominant arteriopathy with subcortical infarcts and leukoencephalopathy
ECM: Extra-cellular matrix
EDS: Ehlers–Danlos syndrome
EMT: Epithelial to mesenchymal transition
ESC: Embryonic stem cell
FACS: Fluorescence-activated cell sorting
FGF: Fibroblast growth factor
GOM: Granular osmiophillic material
HGP: Hutchinson-Gilford progeria
IGF: Insulin-like growth factor
iPSC: induced pluripotent stem cell
LIF: Leukemia inhibitory factor
MACS: Magnetic activated cell sorting
MMP: Matrix metalloproteinase
NCSC: Neural crest stem cell
PDGF-BB: Platelet-derived growth factor-BB
PSC: Pluripotent stem cell
SMC: Smooth muscle cell
TIMP: Tissue inhibitors of MMPs
TGF-β: Transforming growth factor-beta
VEGF: Vascular endothelial growth factor
